# Tetra­kis[μ-*N*-(2,4,6-trimethyl­phen­yl)acetamidato]-κ^4^ *N*:*O*;κ^4^ *O*:*N*-bis­[(benzo­nitrile-κ*N*)rhodium(II)](*Rh*—*Rh*)

**DOI:** 10.1107/S1600536812024518

**Published:** 2012-06-13

**Authors:** Cassandra T. Eagle, Kenneth K. Kpogo, Landon C. Zink, Albert E. Smith

**Affiliations:** a Chemistry Department, East Tennessee State University, PO Box 70695, Johnson City, Tennessee, TN 37614, USA

## Abstract

The title structure, [Rh_2_(C_11_H_14_NO)_4_(C_7_H_5_N)_2_], contains a dinuclear Rh complex of point symmetry -4 with an Rh—Rh unit and two benzonitrile ligands located in special positions along the twofold axis passing through -4. Four symmetry-equivalent mesitylacetamidate ligands bridge the Rh—Rh unit. Thus, each Rh^II^ atom has an approximately octa­hedral coordination by one Rh [Rh—Rh = 2.4290 (6) Å], two acetamidate O atoms *trans* to each other [Rh—O = 2.044 (3) Å], two acetamidate N atoms *trans* to each other [Rh—N = 2.091 (4) Å], and a benzonitrile N atom *trans* to Rh [Rh—N = 2.222 (3) Å]. The structure is held together by weak van der Waals forces.

## Related literature

For the synthesis and crystal structure of a related compound, see: Eagle *et al.* (2000 ▶).
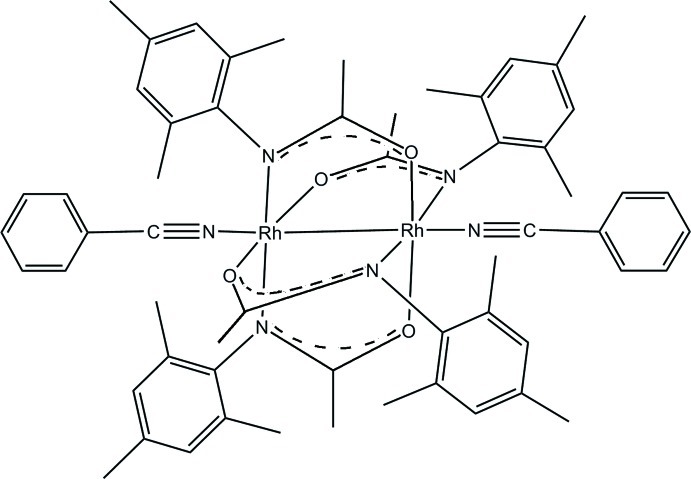


## Experimental

### 

#### Crystal data

[Rh_2_(C_11_H_14_NO)_4_(C_7_H_5_N)_2_]
*M* *_r_* = 1117.01
Tetragonal, 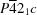

*a* = 10.9928 (19) Å
*c* = 21.4549 (19) Å
*V* = 2592.6 (7) Å^3^
*Z* = 2
Mo *K*α radiation
μ = 0.69 mm^−1^
*T* = 298 K
0.18 × 0.13 × 0.07 mm

#### Data collection

Rigaku XtaLAB mini diffractometer
Absorption correction: multi-scan (*REQAB*; Jacobson, 1998 ▶) *T*_min_ = 0.618, *T*_max_ = 0.953
48863 measured reflections
2969 independent reflections
1950 reflections with *I* > 2σ(*I*)
*R*_int_ = 0.105

#### Refinement

*R*[*F* ^2^ > 2σ(*F* ^2^)] = 0.036
*wR*(*F* ^2^) = 0.074
*S* = 1.03
2969 reflections
165 parameters
H-atom parameters constrained
Δρ_max_ = 0.64 e Å^−3^
Δρ_min_ = −0.71 e Å^−3^
Absolute structure: Flack (1983 ▶), 1275 Friedel pairs
Flack parameter: −0.03 (5)

### 

Data collection: *CrystalClear* (Rigaku, 2011 ▶); cell refinement: *CrystalClear*; data reduction: *CrystalClear*; program(s) used to solve structure: *SIR2004* (Burla *et al.*, 2005 ▶); program(s) used to refine structure: *SHELXL97* (Sheldrick, 2008 ▶); molecular graphics: *CrystalStructure* (Rigaku, 2010 ▶); software used to prepare material for publication: *CrystalStructure*.

## Supplementary Material

Crystal structure: contains datablock(s) global, I. DOI: 10.1107/S1600536812024518/qk2033sup1.cif

Structure factors: contains datablock(s) I. DOI: 10.1107/S1600536812024518/qk2033Isup2.hkl

Additional supplementary materials:  crystallographic information; 3D view; checkCIF report

## Figures and Tables

**Fig. 1.**
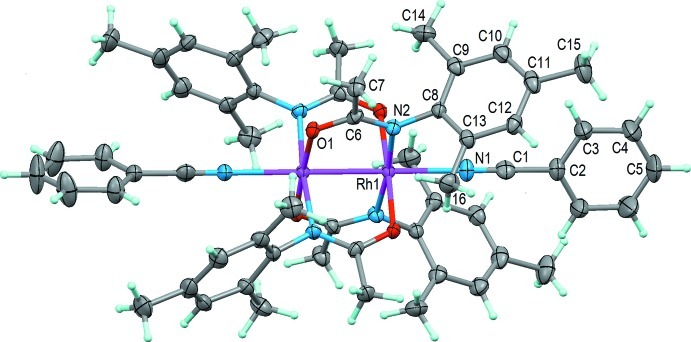
The molecular structure of the title compound showing displacement ellipsoids at the 30% probability level. Hydrogen atoms are shown as small spheres.

**Fig. 2.**
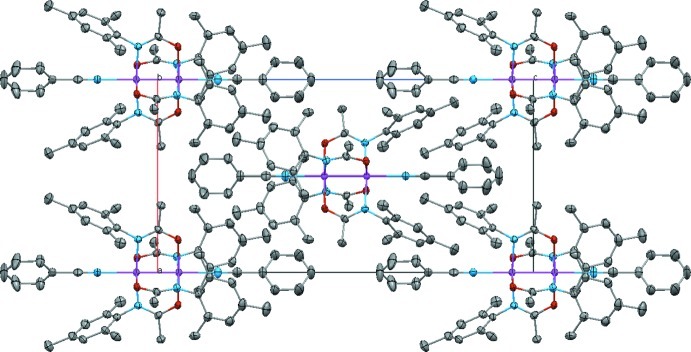
Packing diagram of the title structure viewed along the a-axis. H-atoms omitted for clarity.

## supplementary crystallographic information

### Comment

The title compound, a dinuclear Rh complex with a Rh—Rh bond and four equivalent bridging ligands (Fig. 1), is the mesitylacetamidato analogue of the previously published phenylacetamidato compound Rh_2_[N(C_6_H_5_)C(O)CH_3_]_4_^.^2NCC_6_H_5_ (Eagle *et al.*, 2000), both having the 2,2-*trans* stereochemistry in the complex-core, which is one out of four possible isomers. The highest molecular symmetry that these two complexes can adopt is 42*m* with two mirror planes trough the acetamidate chelate ring pairs and twofold axes in bisecting directions. While in the crystal structure of Rh_2_[N(C_6_H_5_)C(O)CH_3_]_4_^.^2NCC_6_H_5_ the Rh complex has point symmetry 1 and adopts a considerably twisted conformation in the core and one benzonitrile ligand (significantly bent off from the Rh—Rh axis), the complex of the title structure is more regular and has 4 symmetry, not far from 42*m* if the axial benzonitrile ligands are disregarded. Thus, in Rh_2_[N(C_6_H_5_)C(O)CH_3_]_4_^.^2NCC_6_H_5_ the four N—Rh—Rh—O dihedral angles range from 9.03 to 11.89°, while in the title compound the same torsion angle is only 1.12 (9)°. The inclination angle of the mesityl phenyl rings to the Rh—Rh-bond is in the title compound about 26°, while about 34° in the phenylacetamidato complex. This makes the space between adjacent mesityl rings narrow and forces the benzonitrile phenyl rings into a more inclined orientation than in Rh_2_[N(C_6_H_5_)C(O)CH_3_]_4_^.^2NCC_6_H_5_, where they are not far from perpendicular to the acetamidinato phenyl rings. A packing diagram of the structure is shown in Fig. 2. It reveals that the molecules are held together by weak Van der Waals forces, which is not surprising in view of the 16 CH_3_ groups on the outer surface of the Rh complex.

### Experimental

Approximately 10 mg of 2,2-*trans*-tetrakis[µ-(*N*-{2,4,6-trimethylphenyl}acetamidato-κ*N*:κ*O*)]dirhodium(II), synthesized by adapting the procedure described by Eagle *et al.* (2000), was dissolved in 5 mL of dichloromethane forming a green solution. Approximately 2.29 µL of benzonitrile was added to the solution causing the color to become blue. Crystals of the title compound were obtained using a vapor diffusion technique with acetonitrile for a week. The crystal was measured at 298 K on a Rigaku XtaLAB mini diffractometer.

### Refinement

All H atoms were placed in calculated positions and thereafter treated as riding, C—H = 0.93 – 0.96 Å. *U*_ĩso_(H) = 1.2*U*_eq_(C) for CH groups; *U*_ĩso_(H) = 1.5*U*_eq_(C) for CH_3_ groups. A torsional parameter was refined for the methyl groups.

### Crystal data

**Table:**

[Rh_2_(C_11_H_14_NO)_4_(C_7_H_5_N)_2_]	*D*_x_ = 1.431 Mg m^−^^3^
*M**_r_* = 1117.01	Mo *K*α radiation, λ = 0.71075 Å
Tetragonal, *P*42_1_*c*	Cell parameters from 32577 reflections
Hall symbol: P -4 2n	θ = 3.2–27.7°
*a* = 10.9928 (19) Å	µ = 0.69 mm^−^^1^
*c* = 21.4549 (19) Å	*T* = 298 K
*V* = 2592.6 (7) Å^3^	Prism, blue
*Z* = 2	0.18 × 0.13 × 0.07 mm
*F*(000) = 1156.00	

### Data collection

**Table:**

Rigaku XtaLAB mini diffractometer	2969 independent reflections
Radiation source: fine-focus sealed tube	1950 reflections with *I* > 2σ(*I*)
Graphite monochromator	*R*_int_ = 0.105
Detector resolution: 6.827 pixels mm^-1^	θ_max_ = 27.5°
ω scans	*h* = −14→14
Absorption correction: multi-scan (REQAB; Jacobson, 1998)	*k* = −14→14
*T*_min_ = 0.618, *T*_max_ = 0.953	*l* = −27→27
48863 measured reflections	

### Refinement

**Table:**

Refinement on *F*^2^	Secondary atom site location: difference Fourier map
Least-squares matrix: full	Hydrogen site location: inferred from neighbouring sites
*R*[*F*^2^ > 2σ(*F*^2^)] = 0.036	H-atom parameters constrained
*wR*(*F*^2^) = 0.074	*w* = 1/[σ^2^(*F*_o_^2^) + (0.0213*P*)^2^ + 2.1713*P*] where *P* = (*F*_o_^2^ + 2*F*_c_^2^)/3
*S* = 1.03	(Δ/σ)_max_ = 0.003
2969 reflections	Δρ_max_ = 0.64 e Å^−^^3^
165 parameters	Δρ_min_ = −0.71 e Å^−^^3^
0 restraints	Absolute structure: Flack (1983), 1275 Friedel pairs
Primary atom site location: structure-invariant direct methods	Flack parameter: −0.03 (5)

### Special details

**Table:**

Refinement. Refinement was performed using all reflections. The weighted *R*-factor (*wR*) and goodness of fit (*S*) are based on *F*^2^. *R*-factor (gt) are based on *F*. The threshold expression of *F*^2^ > 2.0 σ(*F*^2^) is used only for calculating *R*-factor (gt).

### Fractional atomic coordinates and isotropic or equivalent isotropic displacement parameters (Å^2^)

**Table:**

	*x*	*y*	*z*	*U*_iso_*/*U*_eq_	
Rh1	0.5000	0.5000	0.556606 (14)	0.03097 (10)	
O1	0.6695 (3)	0.4236 (3)	0.44533 (15)	0.0342 (8)	
N1	0.5000	0.5000	0.66016 (16)	0.0443 (9)	
N2	0.6716 (3)	0.4187 (3)	0.55161 (19)	0.0349 (10)	
C1	0.5000	0.5000	0.7117 (2)	0.0490 (12)	
C2	0.5000	0.5000	0.7799 (2)	0.0477 (11)	
C3	0.5590 (5)	0.4097 (5)	0.8123 (2)	0.0669 (16)	
H3	0.6002	0.3484	0.7913	0.080*	
C4	0.5561 (7)	0.4115 (6)	0.8766 (2)	0.100 (2)	
H4	0.5943	0.3493	0.8986	0.119*	
C5	0.5000	0.5000	0.9084 (3)	0.118 (3)	
H5	0.5000	0.5000	0.9517	0.141*	
C6	0.7216 (3)	0.3988 (3)	0.4977 (3)	0.0376 (8)	
C7	0.8472 (3)	0.3448 (4)	0.4909 (2)	0.0481 (11)	
H7A	0.8635	0.3293	0.4476	0.072*	
H7B	0.8516	0.2699	0.5138	0.072*	
H7C	0.9064	0.4009	0.5069	0.072*	
C8	0.7316 (4)	0.3860 (4)	0.60846 (17)	0.0350 (9)	
C9	0.7176 (4)	0.2700 (4)	0.63427 (19)	0.0447 (10)	
C10	0.7689 (4)	0.2470 (5)	0.6922 (2)	0.0538 (12)	
H10	0.7613	0.1693	0.7089	0.065*	
C11	0.8302 (5)	0.3333 (6)	0.7260 (2)	0.0585 (15)	
C12	0.8474 (4)	0.4461 (5)	0.6986 (2)	0.0473 (12)	
H12	0.8909	0.5050	0.7203	0.057*	
C13	0.8015 (4)	0.4747 (4)	0.63927 (18)	0.0416 (11)	
C14	0.6525 (5)	0.1711 (4)	0.5992 (2)	0.0578 (14)	
H14A	0.6895	0.1608	0.5590	0.087*	
H14B	0.5685	0.1931	0.5940	0.087*	
H14C	0.6578	0.0963	0.6221	0.087*	
C15	0.8820 (5)	0.3097 (6)	0.7897 (2)	0.080 (2)	
H15A	0.8394	0.2431	0.8088	0.120*	
H15B	0.8729	0.3812	0.8150	0.120*	
H15C	0.9667	0.2897	0.7861	0.120*	
C16	0.8312 (5)	0.5947 (4)	0.6108 (2)	0.0585 (15)	
H16A	0.7604	0.6459	0.6117	0.088*	
H16B	0.8567	0.5830	0.5685	0.088*	
H16C	0.8955	0.6326	0.6340	0.088*	

### Atomic displacement parameters (Å^2^)

**Table:**

	*U*^11^	*U*^22^	*U*^33^	*U*^12^	*U*^13^	*U*^23^
Rh1	0.0331 (3)	0.0354 (3)	0.02438 (14)	0.0012 (5)	0.000	0.000
O1	0.036 (2)	0.041 (2)	0.0255 (18)	0.0021 (19)	0.0032 (18)	−0.0047 (18)
N1	0.037 (4)	0.066 (5)	0.0301 (19)	−0.006 (7)	0.000	0.000
N2	0.031 (2)	0.041 (3)	0.033 (2)	0.006 (2)	−0.002 (2)	−0.003 (2)
C1	0.037 (4)	0.057 (5)	0.053 (3)	0.014 (7)	0.000	0.000
C2	0.039 (4)	0.068 (6)	0.035 (2)	−0.002 (10)	0.000	0.000
C3	0.093 (4)	0.062 (4)	0.045 (3)	0.025 (3)	−0.003 (3)	0.000 (2)
C4	0.155 (7)	0.086 (5)	0.057 (3)	0.049 (4)	−0.030 (4)	0.005 (3)
C5	0.206 (13)	0.116 (9)	0.030 (3)	0.033 (15)	0.000	0.000
C6	0.0387 (19)	0.0369 (18)	0.0373 (19)	0.0014 (16)	0.010 (3)	−0.007 (3)
C7	0.038 (2)	0.062 (3)	0.045 (3)	0.0112 (19)	0.003 (2)	−0.006 (3)
C8	0.036 (2)	0.040 (2)	0.029 (2)	0.005 (2)	−0.0011 (17)	−0.0024 (18)
C9	0.037 (3)	0.052 (3)	0.045 (2)	0.0105 (19)	−0.002 (2)	0.005 (2)
C10	0.056 (3)	0.056 (3)	0.050 (2)	0.013 (2)	−0.003 (3)	0.008 (3)
C11	0.049 (3)	0.084 (4)	0.042 (3)	0.025 (3)	−0.003 (2)	0.007 (3)
C12	0.039 (3)	0.060 (3)	0.043 (2)	0.007 (2)	−0.012 (2)	−0.011 (2)
C13	0.037 (2)	0.049 (3)	0.039 (2)	0.0027 (19)	−0.0053 (18)	0.003 (2)
C14	0.065 (4)	0.037 (3)	0.072 (3)	0.003 (3)	0.001 (3)	0.004 (3)
C15	0.072 (4)	0.121 (6)	0.048 (3)	0.010 (4)	−0.017 (3)	0.016 (4)
C16	0.057 (4)	0.051 (3)	0.068 (3)	−0.008 (3)	−0.018 (3)	−0.004 (3)

### Geometric parameters (Å, º)

**Table:**

Rh1—O1^i^	2.044 (3)	C7—H7B	0.9600
Rh1—O1^ii^	2.044 (3)	C7—H7C	0.9600
Rh1—N2	2.091 (4)	C8—C9	1.399 (6)
Rh1—N2^iii^	2.091 (4)	C8—C13	1.406 (5)
Rh1—N1	2.222 (3)	C9—C10	1.389 (6)
Rh1—Rh1^i^	2.4290 (6)	C9—C14	1.504 (7)
O1—C6	1.290 (6)	C10—C11	1.370 (7)
O1—Rh1^i^	2.044 (3)	C10—H10	0.9300
N1—C1	1.106 (6)	C11—C12	1.385 (7)
N2—C6	1.300 (6)	C11—C15	1.504 (6)
N2—C8	1.432 (5)	C12—C13	1.406 (5)
C1—C2	1.463 (6)	C12—H12	0.9300
C2—C3^iii^	1.375 (5)	C13—C16	1.489 (6)
C2—C3	1.375 (5)	C14—H14A	0.9600
C3—C4	1.380 (7)	C14—H14B	0.9600
C3—H3	0.9300	C14—H14C	0.9600
C4—C5	1.338 (6)	C15—H15A	0.9600
C4—H4	0.9300	C15—H15B	0.9600
C5—C4^iii^	1.338 (6)	C15—H15C	0.9600
C5—H5	0.9300	C16—H16A	0.9600
C6—C7	1.510 (5)	C16—H16B	0.9600
C7—H7A	0.9600	C16—H16C	0.9600
			
O1^i^—Rh1—O1^ii^	177.67 (18)	C6—C7—H7C	109.5
O1^i^—Rh1—N2	88.85 (16)	H7A—C7—H7C	109.5
O1^ii^—Rh1—N2	91.03 (16)	H7B—C7—H7C	109.5
O1^i^—Rh1—N2^iii^	91.03 (16)	C9—C8—C13	120.4 (4)
O1^ii^—Rh1—N2^iii^	88.85 (16)	C9—C8—N2	121.0 (4)
N2—Rh1—N2^iii^	174.1 (2)	C13—C8—N2	118.5 (4)
O1^i^—Rh1—N1	91.16 (9)	C10—C9—C8	118.4 (5)
O1^ii^—Rh1—N1	91.16 (9)	C10—C9—C14	120.7 (4)
N2—Rh1—N1	92.94 (11)	C8—C9—C14	120.9 (4)
N2^iii^—Rh1—N1	92.94 (11)	C11—C10—C9	123.2 (5)
O1^i^—Rh1—Rh1^i^	88.84 (9)	C11—C10—H10	118.4
O1^ii^—Rh1—Rh1^i^	88.84 (9)	C9—C10—H10	118.4
N2—Rh1—Rh1^i^	87.06 (11)	C10—C11—C12	117.6 (4)
N2^iii^—Rh1—Rh1^i^	87.06 (11)	C10—C11—C15	123.2 (6)
N1—Rh1—Rh1^i^	180.0	C12—C11—C15	119.2 (6)
C6—O1—Rh1^i^	120.6 (3)	C11—C12—C13	122.4 (5)
C1—N1—Rh1	180.000 (1)	C11—C12—H12	118.8
C6—N2—C8	121.4 (4)	C13—C12—H12	118.8
C6—N2—Rh1	119.9 (3)	C12—C13—C8	117.8 (4)
C8—N2—Rh1	118.6 (3)	C12—C13—C16	119.4 (4)
N1—C1—C2	180.000 (2)	C8—C13—C16	122.8 (4)
C3^iii^—C2—C3	119.2 (5)	C9—C14—H14A	109.5
C3^iii^—C2—C1	120.4 (3)	C9—C14—H14B	109.5
C3—C2—C1	120.4 (3)	H14A—C14—H14B	109.5
C2—C3—C4	119.0 (5)	C9—C14—H14C	109.5
C2—C3—H3	120.5	H14A—C14—H14C	109.5
C4—C3—H3	120.5	H14B—C14—H14C	109.5
C5—C4—C3	122.0 (5)	C11—C15—H15A	109.5
C5—C4—H4	119.0	C11—C15—H15B	109.5
C3—C4—H4	119.0	H15A—C15—H15B	109.5
C4—C5—C4^iii^	118.8 (6)	C11—C15—H15C	109.5
C4—C5—H5	120.6	H15A—C15—H15C	109.5
C4^iii^—C5—H5	120.6	H15B—C15—H15C	109.5
O1—C6—N2	123.5 (3)	C13—C16—H16A	109.5
O1—C6—C7	113.9 (4)	C13—C16—H16B	109.5
N2—C6—C7	122.6 (5)	H16A—C16—H16B	109.5
C6—C7—H7A	109.5	C13—C16—H16C	109.5
C6—C7—H7B	109.5	H16A—C16—H16C	109.5
H7A—C7—H7B	109.5	H16B—C16—H16C	109.5
			
O1—Rh1^i^—Rh1—N2	−1.10 (16)	Rh1—N2—C8—C9	91.8 (4)
O1^i^—Rh1—N2—C6	90.8 (3)	C6—N2—C8—C13	94.2 (5)
O1^ii^—Rh1—N2—C6	−86.9 (3)	Rh1—N2—C8—C13	−85.8 (4)
N1—Rh1—N2—C6	−178.1 (3)	C13—C8—C9—C10	3.2 (6)
Rh1^i^—Rh1—N2—C6	1.9 (3)	N2—C8—C9—C10	−174.3 (4)
O1^i^—Rh1—N2—C8	−89.2 (3)	C13—C8—C9—C14	−174.6 (4)
O1^ii^—Rh1—N2—C8	93.1 (3)	N2—C8—C9—C14	7.9 (6)
N1—Rh1—N2—C8	1.9 (3)	C8—C9—C10—C11	1.6 (7)
Rh1^i^—Rh1—N2—C8	−178.1 (3)	C14—C9—C10—C11	179.4 (5)
C3^iii^—C2—C3—C4	0.8 (5)	C9—C10—C11—C12	−4.1 (7)
C1—C2—C3—C4	−179.2 (5)	C9—C10—C11—C15	177.8 (5)
C2—C3—C4—C5	−1.6 (10)	C10—C11—C12—C13	1.9 (7)
C3—C4—C5—C4^iii^	0.8 (5)	C15—C11—C12—C13	−179.9 (4)
Rh1^i^—O1—C6—N2	0.6 (4)	C11—C12—C13—C8	2.6 (7)
Rh1^i^—O1—C6—C7	−179.0 (2)	C11—C12—C13—C16	−175.6 (5)
C8—N2—C6—O1	178.0 (4)	C9—C8—C13—C12	−5.2 (6)
Rh1—N2—C6—O1	−2.0 (5)	N2—C8—C13—C12	172.4 (4)
C8—N2—C6—C7	−2.4 (6)	C9—C8—C13—C16	172.9 (4)
Rh1—N2—C6—C7	177.6 (3)	N2—C8—C13—C16	−9.5 (6)
C6—N2—C8—C9	−88.2 (5)		

Symmetry codes: (i) *y*, −*x*+1, −*z*+1; (ii) −*y*+1, *x*, −*z*+1; (iii) −*x*+1, −*y*+1, *z*.
